# Unveiling the improved targeting migration of mesenchymal stem cells with CXC chemokine receptor 3-modification using intravital NIR-II photoacoustic imaging

**DOI:** 10.1186/s12951-022-01513-7

**Published:** 2022-06-28

**Authors:** Yuejun Lin, Hui-chao Zhou, Ningbo Chen, Yaguang Ren, Rongkang Gao, Qiaojia Li, Yiwen Deng, Xuejiao Han, Xiaoran Zhang, Andy Peng Xiang, Bing Guo, Chengbo Liu, Jie Ren

**Affiliations:** 1 Department of Ultrasound, Laboratory of Novel Optoacoustic/Ultrasonic Imaging, Key Laboratory of Liver Disease of Guangdong Province, The Third Affiliated Hospital, Sun Yat-Sen University, Guangzhou, 510630 China; 2 Research Laboratory for Biomedical Optics and Molecular Imaging, Shenzhen Institutes of Advanced Technology, Chinese Academy of Sciences, Shenzhen, 518055 China; 3 School of Science and Shenzhen Key Laboratory of Flexible Printed Electronics Technology, Harbin Institute of Technology, Shenzhen, 518055 China; 4 Center for Stem Cell Biology and Tissue Engineering, Key Laboratory for Stem Cells and Tissue Engineering, Ministry of Education, Sun Yat-Sen University, Guangzhou, 510630 China; 5 Department of Medical Oncology, Harbin Medical University Cancer Hospital, Harbin Medical University, Harbin, 150081 China

**Keywords:** Photoacoustic imaging, Second near-infrared window, Mesenchymal stem cell, Targeting migration, Gene modification, Conjugated polymer nanoparticles

## Abstract

**Background:**

Therapy with genetically modified mesenchymal stem cells (MSCs) has clinical translation promise. Optimizing the targeting migratory ability of MSCs relies on accurate imaging of the distribution and extravasation kinetics of MSCs, and the corresponding imaging results could be used to predict therapeutic outcomes and guide the optimization of the treatment program. Among the different imaging modalities, second near-infrared (NIR-II) optical-resolution photoacoustic microscopy (OR-PAM) has merits, including a fine resolution, a deep penetration, a high sensitivity, and a large signal-to-background ratio. It would be an ideal candidate for precise monitoring of MSCs, although it has not been tested for this purpose so far.

**Results:**

Penetrating peptide-decorated conjugated polymer nanoparticles (TAT-CPNPs) with strong NIR-II absorbance were used to label chemokine-receptor genetically modified MSCs, which were subsequently evaluated under intravital NIR-II OR-PAM regarding their targeting migratory ability. Based on the upregulation of chemokine (C-X-C motif) ligand 10 in the inflamed ears of contact hypersensitivity mice, MSCs with overexpression of corresponding receptor, chemokine (C-X-C motif) receptor 3 (Cxcr3) were successfully generated (MSC^Cxcr3^). TAT-CPNPs labeling enabled NIR-II photoacoustic imaging to discern MSC^Cxcr3^ covered by 1.2 cm of chicken breast tissue. Longitudinal OR-PAM imaging revealed enhanced inflammation-targeting migration of MSC^Cxcr3^ over time attributed to Cxcr3 gene modification, which was further validated by histological analysis.

**Conclusions:**

TAT-CPNPs-assisted NIR-II PA imaging is promising for monitoring distribution and extravasation kinetics of MSCs, which would greatly facilitate optimizing MSC-based therapy.

**Graphical Abstract:**

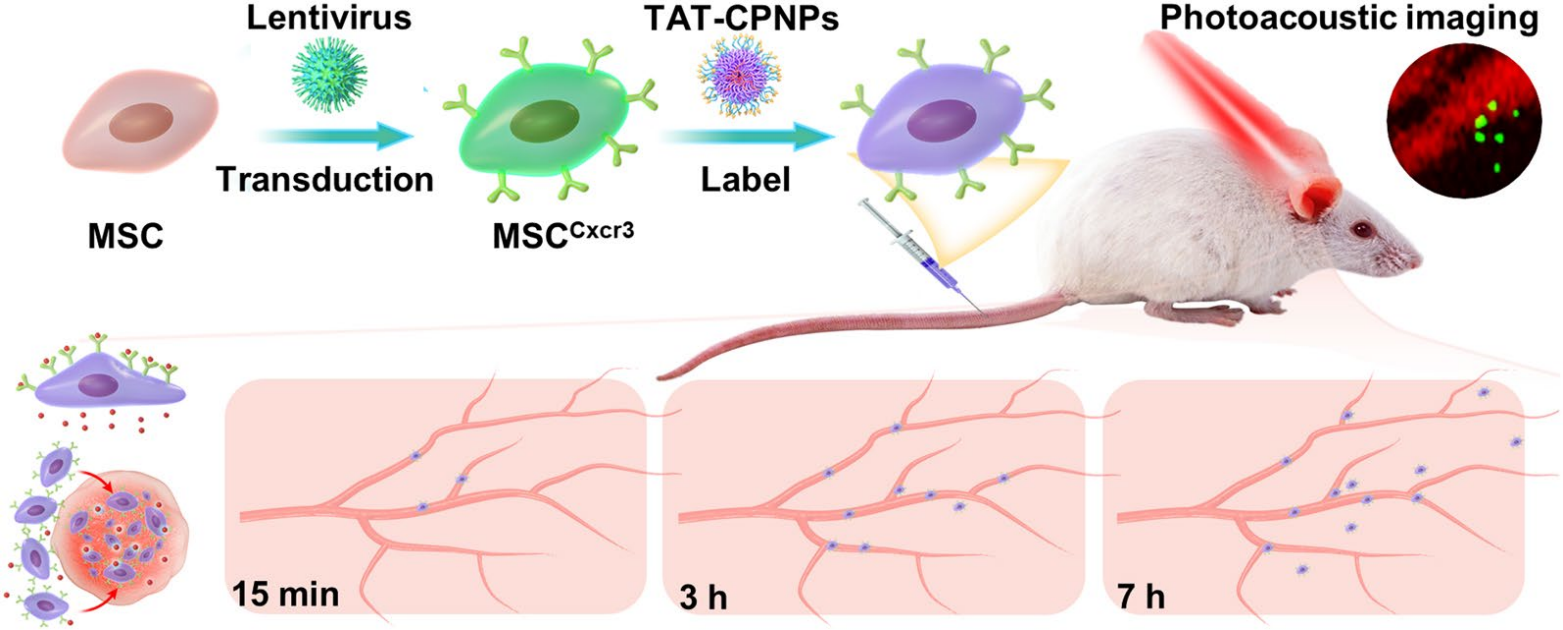

**Supplementary Information:**

The online version contains supplementary material available at 10.1186/s12951-022-01513-7.

## Background

By virtue of their multipotent, trophic, and immunomodulatory properties, mesenchymal stem cells (MSCs) demonstrate great therapeutic potential for treating various refractory diseases, such as bone fracture malignancies, autoimmune disorders, and dermatitis, etc. [1–6]. While local transplantation of MSCs can promote local tissue healing, systemic transplantation of MSCs is of particular significance in treating systemic diseases. However, currently, the therapeutic efficacy for most cases of MSC treatment is unsatisfactory because most of the MSCs are distributed in normal organs and the density of MSC is inadequate at lesions [7].

To overcome this limitation, strategies that modify MSCs with cell trafficking-related genes have been explored to improve their targeting migratory ability. For example, by exploiting the chemotactic response to chemokines released from lesions, we and others reported enhanced targeting migration and improved MSC therapeutics by overexpressing corresponding chemokine receptors in MSCs [8, 9]. Most relevant studies performed histological staining of MSCs in tissue sections of interest to evaluate their targeting migratory capacity. However, this invasive assessment could only be conducted at a single time point. Consequently, information about the location and quantification of MSCs could not be accessed dynamically or continuously. This impedes the comprehensive understanding on their migratory behavior and an objective analysis of the effect of gene modification on the targeting migration of MSCs, this as well prevents imaging guided prediction of therapeutic outcomes and optimization of the treatment program [10]. Therefore, it is essential to conduct a longitudinal comparison of targeting migratory potency between normal and modified MSCs, which would be of great help for potential clinical translation.

To optimize MSC modification for MSC therapy, we should precisely and in time monitor the distribution of MSCs in individuals [11]. Although various conventional imaging modalities have been explored for in vivo MSC imaging, they all have certain disadvantages [12]. For instance, the application of positron emission tomography and single photon emission computing tomography are hindered by low spatial resolution, radiation hazards and high cost, albeit with ultrasensitive detection [13, 14]. To date, optical imaging such as fluorescence and bioluminescence imaging, with intrinsically high sensitivity, has been employed in discerning factors facilitating the targeting migration of MSCs [15–17]. However, conventional optical imaging methods suffer from a limited penetration depth of less than 1 mm due to severe light scattering and reflection within biological tissues, defined as the optical diffusion limit [18].

Recently, photoacoustic imaging, as an emerging hybrid imaging modality which works with both optical light excitation and ultrasound detection, has shown superior penetration depth compared to conventional optical imaging, and has been applied in cancer and stem cell imaging [18–20]. Combined with cell labeling, PA imaging, either using PA computed tomography (PACT) or PA microscopy (PAM), has already demonstrated its potential in MSCs tracking [21–23].

Among the typical PA imaging modalities, OR-PAM has the highest resolution and can resolve fine vasculature with the assistance of either endogenous hemoglobin or exogenous contrast agents [24, 25]. Hence, OR-PAM enables delicate analysis of the position of MSCs relative to label-free blood vessels, providing an informative modality to study the course of MSC migration.

However, using OR-PAM for in vivo monitoring of MSCs still faces two challenges. First, endogenous contrast agents may cause background interference, especially when choosing an excitation wavelength shorter than 1000 nm. Comparatively, tissues have a relatively low absorption and scattering coefficient of the NIR-II (1000–1700 nm) light [26]. Recent reports by us and others have demonstrated the superior imaging performance of NIR-II OR-PAM using biocompatible exogenous contrast agents with a high extinction coefficient, showing compelling penetration depth and signal-to-noise ratio for disease diagnosis and dynamic therapeutic monitoring [27, 28]. However, the performance of NIR-II OR-PAM imaging for MSCs imaging has rarely been explored. It is still unknown whether exogenous contrast agent-assisted NIR-II OR-PAM can be employed to track intravenously transplanted MSCs and whether this strategy can be used to assess the effect of gene modification on the targeting migratory ability of MSCs. Therefore, in this study, precise visualization of intravenously delivered MSCs and identification of the effect of chemokine receptor-gene modification on the targeting migratory ability of MSCs in vivo using OR-PAM became our interest.

The delivery efficiency of MSCs to target sites is limited, which demands that the cell labeling agent be biocompatible as well as producing strong PA signal. Previously, we developed organic donor–acceptor (D–A) structured conjugated polymer nanoparticles (CPNPs) for NIR-II PA imaging and found that they generally exhibited large extinction coefficient to diminish noise interference from living tissues and importantly showed good biocompatibility [27, 28].

**Fig. 1:**
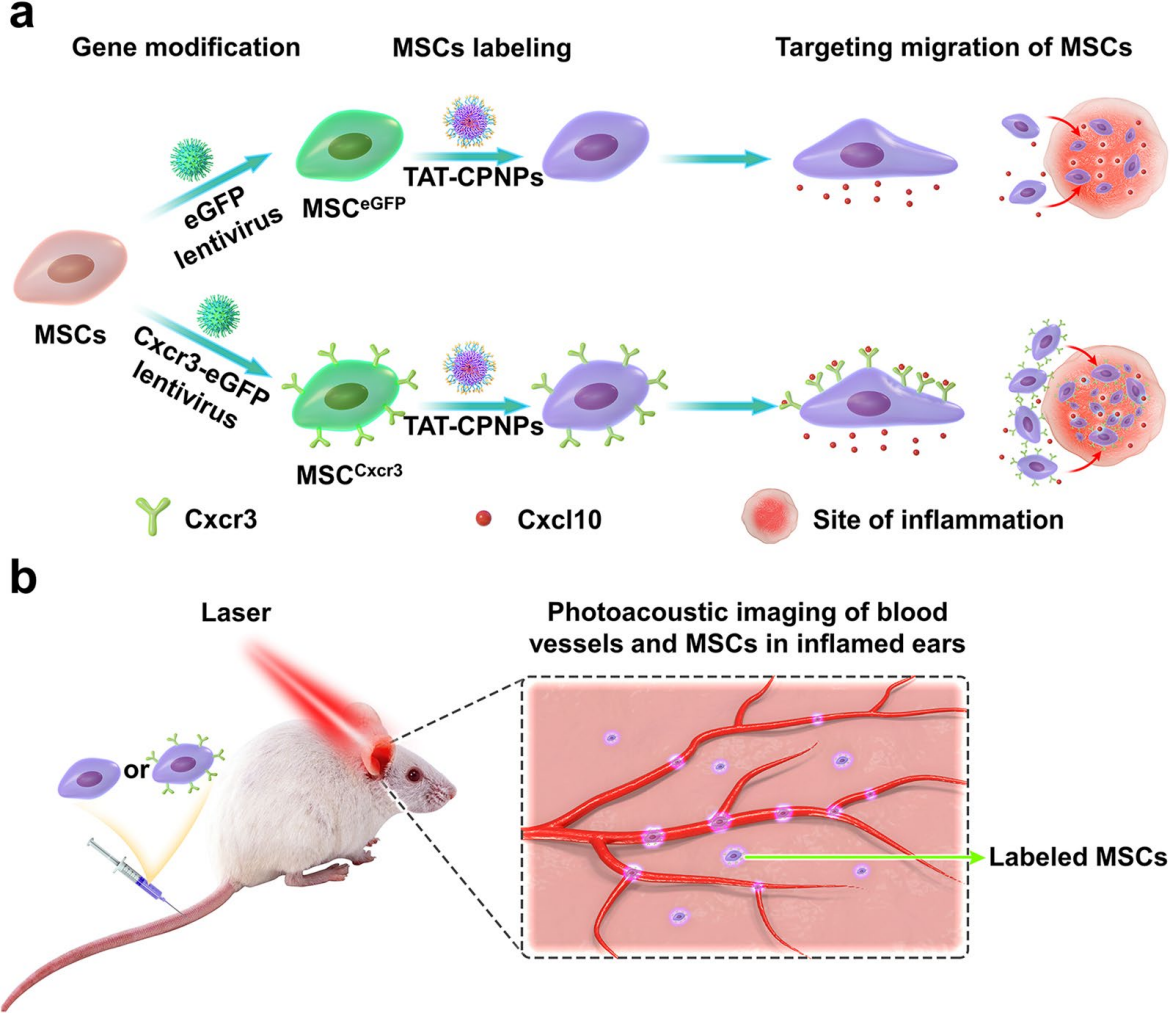
Schematic illustration of experimental design. **a** Labeling of genetically modified MSCs with TAT-CPNPs. Aiming to screen genes that promote the targeting migratory ability of MSCs, Cxcr3, whose ligand was up-regulated in the CHS animal model, was selected as a candidate gene and transduced into MSCs with lentivirus. To visualize gene modified MSCs via NIR-II PA imaging, TAT-CPNPs with high absorbance at 1064 nm were fabricated to label modified MSCs. **b** Longitudinal PA imaging of non-inflamed or inflamed ears in the CHS mouse model for assessing the targeting migratory ability of TAT-CPNPs labelled MSCs. TAT-CPNPs labeled MSCs were intravenously delivered to CHS mice, PA imaging at 1064 nm of non-inflamed or inflamed ears were performed at different time points after injection. The accumulation within targeted tissues and extravasation of MSC^eGFP^ and MSC^Cxcr3^ were analyzed

In this study, we report a novel strategy to study the targeting migratory potency of genetically modified MSCs by longitudinal monitoring of CPNPs labeled MSCs in the site of inflammation using NIR-II OR-PAM imaging (Fig. 1). Conjugated polymer poly(thiadiazolobenzotriazole (TBZ) -alt-thiophene-substituted diketopyrrolopyrrole (DPP) (PTD) was synthesized and exhibited strong NIR-II absorbance. To enhance cellular uptake, PTD was further formulated into water dispersible cell-penetrating peptide TAT decorated nanoparticles (TAT-CPNPs) for MSCs labeling. The contact hypersensitivity (CHS) mouse model was established for intravital monitoring. To improve targeting migration toward inflamed tissues of MSCs, we established chemokine (C-X-C motif) receptor 3 (Cxcr3)-overexpressing MSCs (MSC^Cxcr3^) based on the upregulation of chemokine (C-X-C motif) ligand 10 (Cxcl10) in inflammation site [29]. The NIR-II OR-PAM imaging performance of TAT-CPNPs was analyzed by in vitro imaging of TAT-CPNPs labeled MSCs. For in vivo investigation, we delivered TAT-CPNPs labeled MSC^Cxcr3^ and its control MSC^eGFP^ intravenously into CHS mice. Longitudinal NIR-II PA imaging using OR-PAM was performed on the inflamed ears of the CHS model to reveal the targeting migratory tendency of MSCs, which was further validated by histological analysis.

## Results and discussion

### Synthesis and Characterization of TAT-CPNPs

**Fig. 2:**
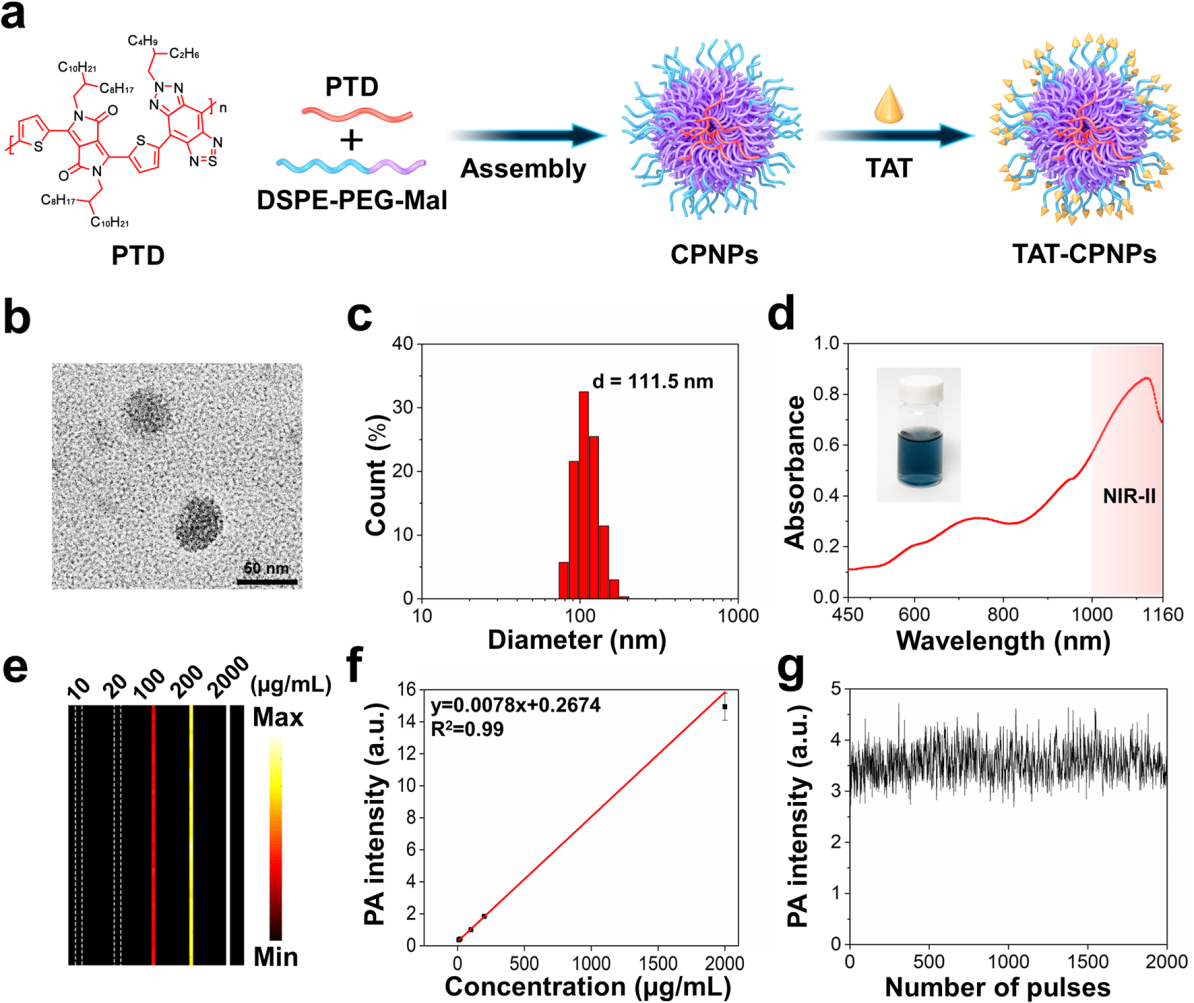
Characterization of TAT-CPNPs. **a** Preparation of TAT-CPNPs. **b** Representative TEM images of TAT-CPNPs. Scale bar, 50 nm. **c** Size distribution of TAT-CPNPs in DW measured with DLS. **d** UV–vis spectra of TAT-CPNPs and photograph (insert) of TAT-CPNPs in DW. **e** PA images of TAT-CPNPs of different concentrations under 1064 nm excitation. **f** The linear fit of PA intensity versus concentration of TAT-CPNPs under 1064 nm excitation. Error bars, SEM. **g** PA intensity of TAT-CPNPs under 2000 continuous 1064 nm pulses excitation

To achieve good fidelities in NIR-II OR-PAM imaging, it is necessary that the contrast agents have characteristics including good biocompatibility, negligible photobleaching effect, suitable particle size and surficial modification. More importantly, the contrast agent should have high NIR-II absorbance to eliminate the background noise from tissue and optimize imaging sensitivity, which is especially critical for imaging of MSCs for the limited amount of MSCs arriving in target tissues. We synthesized the donor–acceptor structured conjugated polymer PTD since it fulfilled the aforementioned requirements; in particular, it was characterized by a quite large extinction coefficient estimated to be 40.1 L g^−1^ cm^−1^ at 1064 nm [27]. To achieve a high in vivo concentration of CPNPs, we performed surface modification with TAT peptide, which is a strong cell-penetrating peptide that enhances cellular uptake. The formulation of TAT-CPNPs is briefly described as follows. First, CPNPs were manufactured by mixing PTD with 1,2-Distearoyl-sn-glycero-3-phosphoethanolamine-*N*-[methoxy(polyethylene glycol)-2000] (DSPE-PEG_2000_) and DSPE-PEG_2000_-maleimide (MAL) by the nanoprecipitation method. Then, to enhance internalization by MSCs, the cell-penetrating peptide TAT was further covalently decorated on the surface of CPNPs via Michael addition reaction to finally fabricate TAT-CPNPs (Fig. 2a) [30]. Approximately 45% of maleimide was linked to TAT determined by BCA protein assay (Additional file 1: Fig. S1a).

As shown in Fig. 2b, c, TAT-CPNPs were spherical and had an average hydrodynamic diameter of around 111.5 nm determined by dynamic light scattering (DLS). As double D-A type conjugated polymer-based NPs, TAT-CPNPs exhibited a broad absorption peak in the NIR-II region (Fig. 2d). Regarding the absorption spectrum of TAT-CPNPs, the commercially available and widely used 1064 nm Nd: YAG laser was chosen for NIR-II PA excitation [19]. TAT-CPNPs of different concentrations were subjected to PA imaging. The PA signal intensity of TAT-CPNPs varied linearly with the concentration (Fig. 2e, f). Meanwhile, the PA signal intensity of TAT-CPNPs was stable during 2000 continuous pulses of 1064 nm laser excitation (Fig. 2g), demonstrating good photostability.

**Fig. 3:**
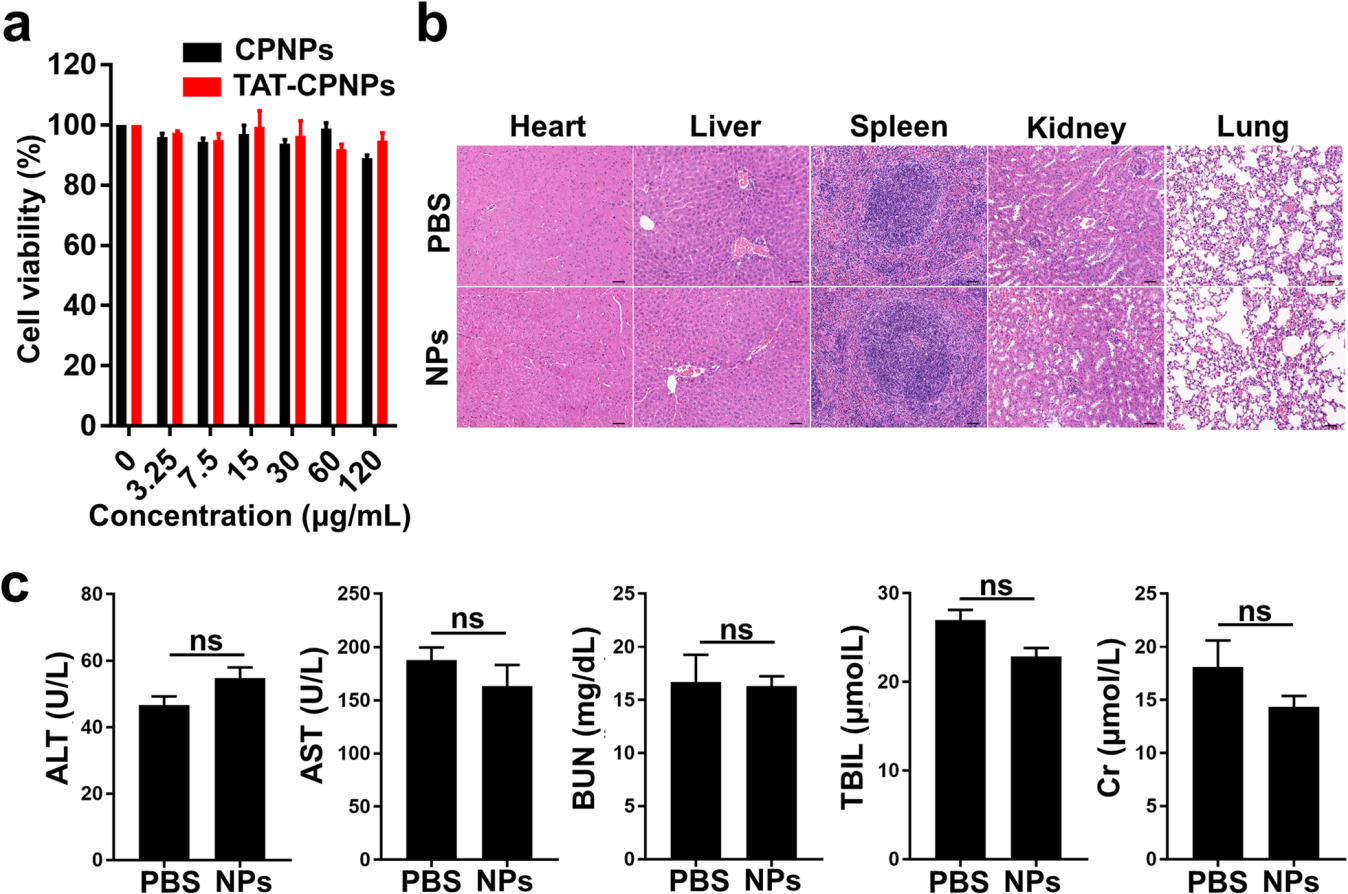
Biosafety of TAT-CPNPs. **a** Cytotoxicity of CPNPs and TAT-CPNPs on MSCs determined by CCK-8 assay. **b** Representative H&E stained sections of major organs collected from mice on day 14 after receiving PBS or TAT-CPNPs injections (n = 3 per group). Scale bars, 50 µm. **c** Analysis of serum biochemistry markers for liver (ALT, AST and TBIL) and kidney (BUN and Cr) on day 14 after mice received PBS or TAT-CPNPs injections (n = 3 per group). ns, no significance; error bars, SEM

Before biological application, the biocompatibility of TAT-CPNPs was evaluated both in vitro and in vivo. In vitro Cell Counting Kit-8 (CCK-8) analysis showed that TAT-CPNPs at a concentration of up to 120 µg/mL demonstrated good biocompatibility with cell viability > 89%, and TAT modification did not change cell viability (Fig. 3a). In vivo histological analysis via hematoxylin and eosin (H&E) staining demonstrated that TAT-CPNPs did not cause significant damage or inflammation in major organs (Fig. 3b). Moreover, in vivo serum biochemistry analysis, including liver function (aspartate aminotransferase (AST), alanine transaminase (ALT), total bilirubin (TBIL)) and kidney function (creatinine (Cr) and blood urea nitrogen (BUN)) analysis, revealed no difference between mice injected with either phosphate buffered saline (PBS) or TAT-CPNPs (Fig. 3c). Collectively, TAT-CPNPs demonstrated good biocompatibility both in vitro and in vivo, which is appealing for potential clinical applications. The abovementioned data demonstrated that TAT-CPNPs with peak optical absorption in the NIR-II window, good biocompatibility and photostability might be a promising NIR-II PA imaging contrast agent.

### Contact hypersensitivity-targeting MSC labeling

#### Cxcl10 was highly upregulated in the inflamed ears of CHS mice

Allergic contact dermatitis (ACD) is a common skin disease caused by a T cell-mediated immune reaction to usually innocuous allergens. Preferred management for ACD is the removal of the offending agents, such as dust mites, but this is not always possible [31, 32]. In those unique situations, immunomodulatory therapies such as MSC transplantation have been proposed, which has benefited severe refractory ACD cases [5, 6]. Hence, we chose the CHS model recapitulating human ACD to explore the feasibility of in vivo MSC tracking with NIR-II PA imaging.

**Fig. 4:**
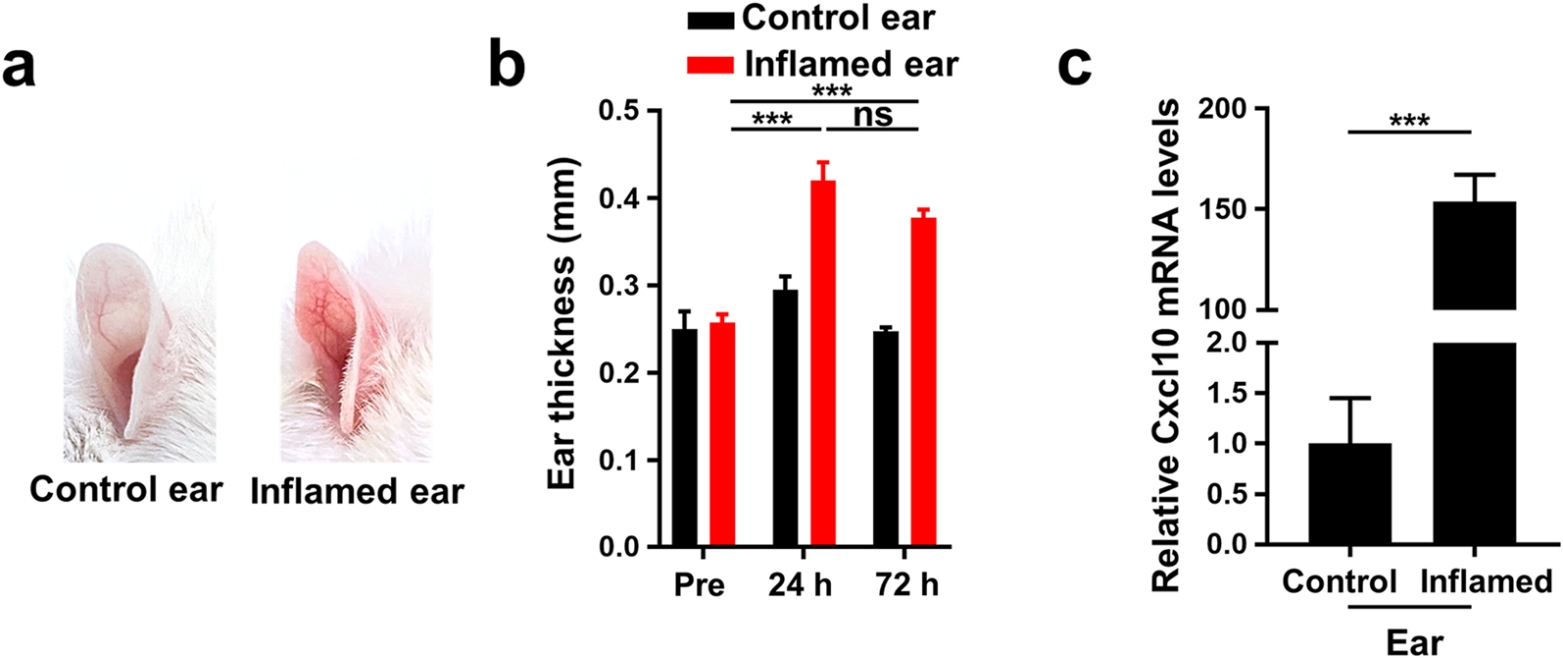
Upregulation of Cxcl10 in inflamed ears of CHS mice. **a** Representative photographs of mouse ears challenged with vehicle (Control) or DNFB (Inflamed). Photos were taken 24 h after challenging. **b** Thickness of mice ears challenged with vehicle (Control) or DNFB (Inflamed) before (Pre), 24 h and 72 h after challenging (n = 3–4 per group). **c** mRNA expression of Cxcl10 transcript in mouse ears challenged with vehicle (Control) or DNFB (Inflamed) (n = 3 per group). ***P < 0.001; ns, no significance; error bars, SEM

To induce CHS, mouse ears were sensitized and challenged with 2,4-dinitro-1-fluorobenzene (DNFB) to cause inflammation, exhibiting redness and swelling. The thickness of the ears increased significantly in the inflamed group compared to the control group (Fig. 4a, b). Consistent with previous findings, Cxcl10 mRNA levels were ~ 150-fold upregulated in inflamed ears of CHS mice compared to control ears (Fig. 4c) [9, 29]. Hence, Cxcr3, the receptor of Cxcl10, was selected to be overexpressed in MSCs to enhance their CHS-targeting migratory ability.

#### Establishment of Cxcr3-overexpressing MSCs

**Fig. 5:**
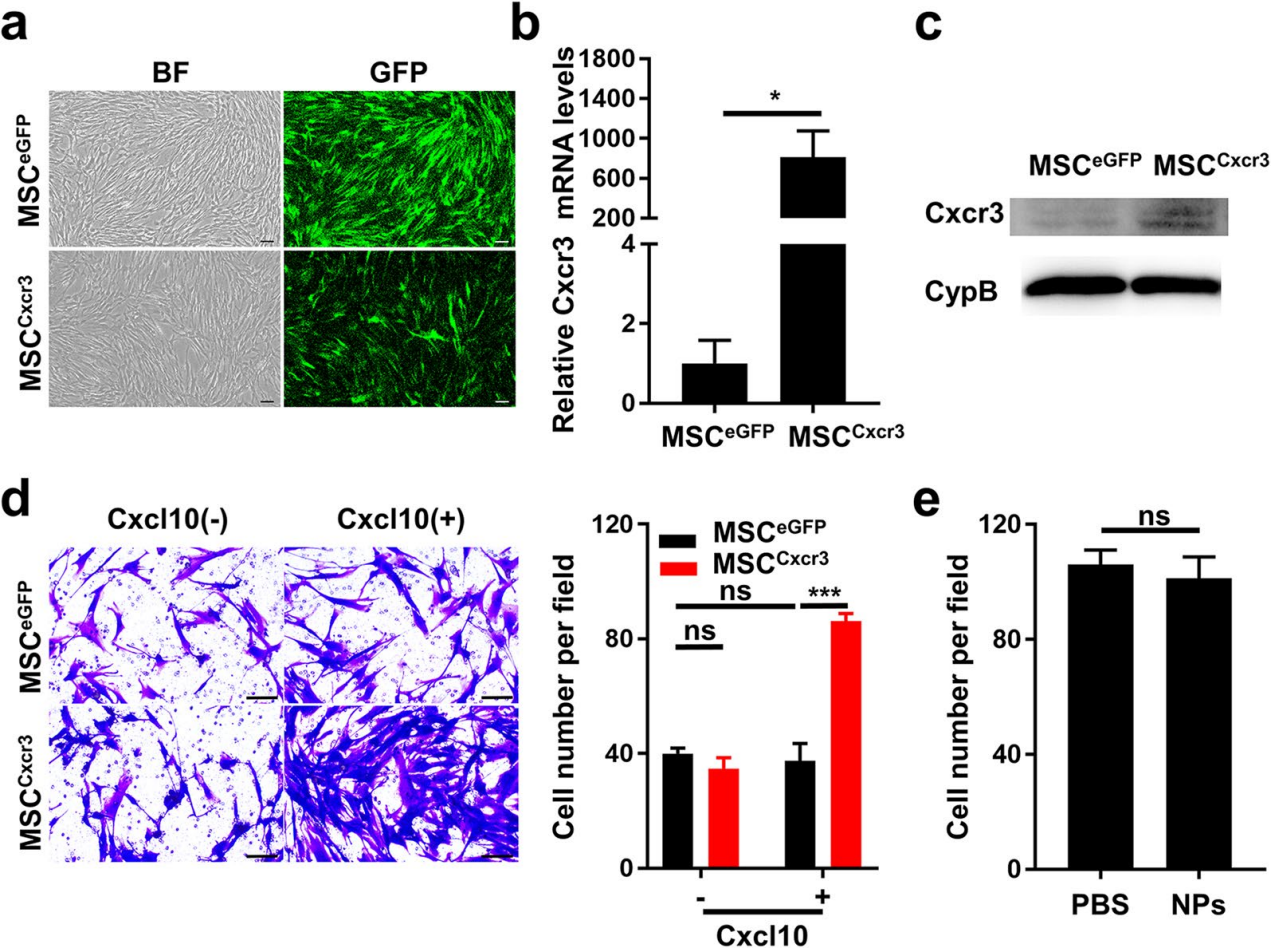
Overexpression of Cxcr3 in MSCs. **a** Representative bright field (BF) and green fluorescence images of MSC^eGFP^ and MSC^Cxcr3^. MSCs were transduced with lentivirus to express eGFP alone (MSC^eGFP^) or in combination with Cxcr3 (MSC^Cxcr3^). **b** Analysis of Cxcr3 mRNA expression in MSC^eGFP^ and MSC^Cxcr3^.**c** Western blot of Cxcr3 in total cell lysates of MSC^eGFP^ and MSC^Cxcr3^. CypB, cyclophilin B, used as housekeeping gene. **d** Cxcr3 overexpression promoted the in vitro migration of MSCs toward Cxcl10. Representative images and quantification analysis of transwell migration assay were shown. **e** Quantification analysis of transwell migration assay of MSC^Cxcr3^ after incubating with vehicle (PBS) or TAT-CPNPs (NPs). Scale bars, 100 µm. *P < 0.05, ***P < 0.001; error bars, SEM

Lentivirus transduction was carried out to establish Cxcr3-overexpressing MSCs. MSCs were successfully transduced with lentiviral vectors encoding enhanced green fluorescence protein (eGFP) + Cxcr3 (referred to as MSC^Cxcr3^) or eGFP (referred to as MSC^eGFP^), as indicated by the expression of the reporter gene eGFP (Fig. 5a and Additional file 1: Fig. S2). Moreover, both the mRNA and protein levels of Cxcr3 were significantly higher in MSC^Cxcr3^ than in MSC^eGFP^ (Fig. 5b-c).

To investigate whether Cxcr3 modification could enhance the migratory ability of MSCs towards its ligand Cxcl10, transwell assays were performed. In the absence of Cxcl10, MSC^Cxcr3^ showed migration ability comparable to that of MSC^eGFP^. Exposure to chemokine Cxcl10 dramatically increased the number of MSC^Cxcr3^ migrated through the membrane but it did not affect the number of migrated MSC^eGFP^ (Fig. 5d), validating the chemotaxis response of MSC^Cxcr3^ in vitro. Uptake of TAT-CPNPs had no significant effect on the chemotactic migration of MSC^Cxcr3^ in vitro (Fig. 5e).

#### In vitro imaging of MSC^Cxcr3^ labeled by TAT-CPNPs

**Fig. 6:**
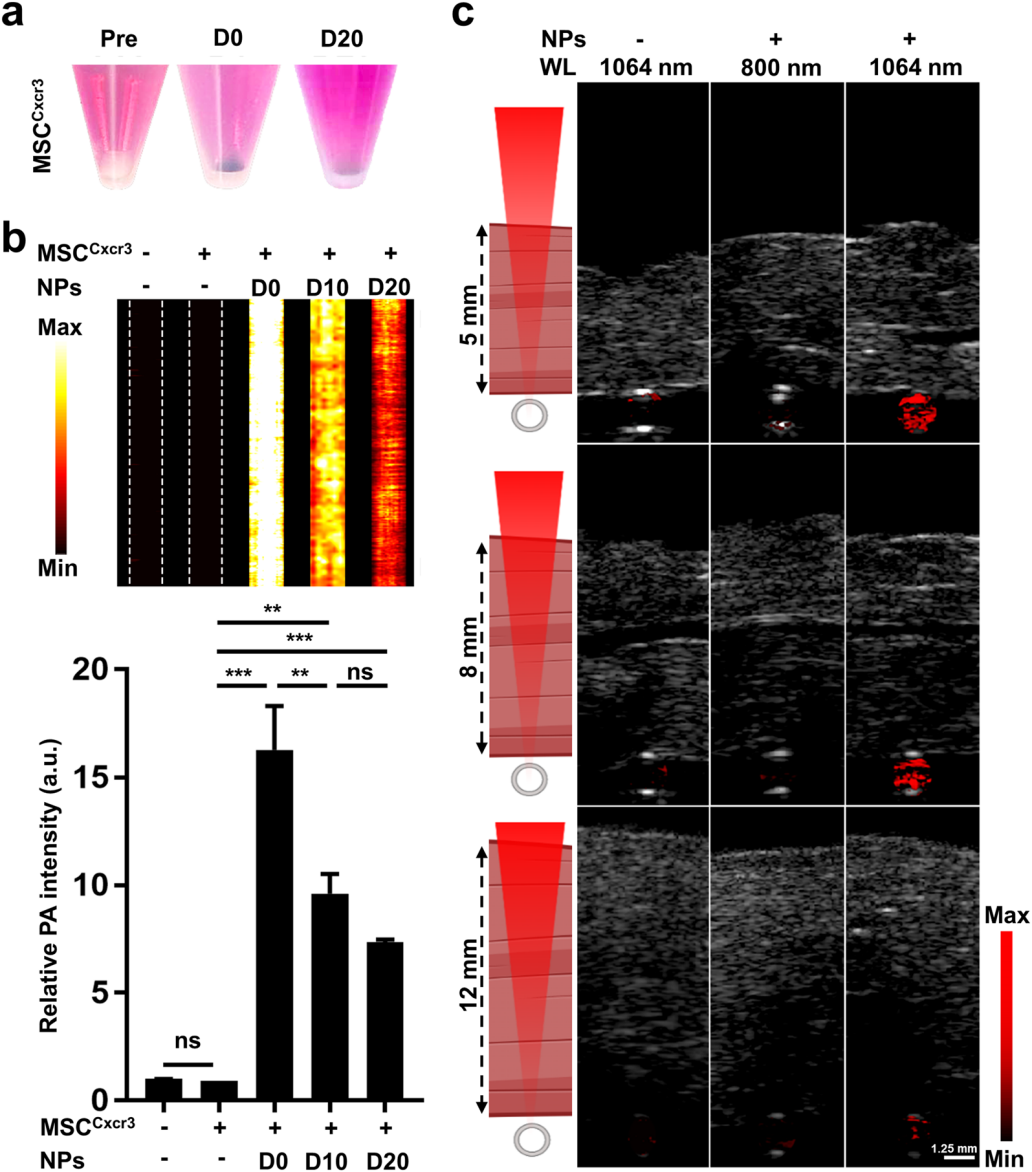
In vitro PA imaging of labeled MSC^Cxcr3^. **a** Photographs of MSC^Cxcr3^ before (Pre), after (D0) and 20 days after (D20) uptake of TAT-CPNPs, respectively. **b** In vitro long-term PA tracking of TAT-CPNPs labeled MSC^Cxcr3^. PA imaging under 1064 nm excitation were performed for tube phantoms containing PBS (lane 1), unlabeled MSC^Cxcr3^ (lane 2) and TAT-CPNPs labeled MSC^Cxcr3^ collected right after (D0, lane 3), 10 days (D10, lane 4) or 20 days after (D20, lane 5) incubation with TAT-CPNPs. Representative PA images and quantification analysis were shown. **c** Penetration performance assessment of NIR-II PA imaging for deep tissue located-labeled MSC^Cxcr3^ resuspended in PBS at 2 × 10^6^ cells/mL. PA and US overlaid images showed PA signal of unlabeled or labeled MSC^Cxcr3^ covered by different thickness of chicken breast tissue. PA images were acquired under 800 nm or 1064 nm laser excitation. Only the tube region of interest is shown for the PA image. Scale bar, 1.25 mm. WL, wavelength. ns, no significance, **P < 0.01, ***P < 0.001; error bars, SEM

After uptake of TAT-CPNPs, the MSC^Cxcr3^ pellet turned dark (Fig. 6a). TAT modification enhanced the PA intensity of labeled MSCs (Additional file 1: Fig. S1b). MSC^Cxcr3^ were cultured with TAT-CPNPs for 16 h for the following in vitro and in vivo PA imaging. The labeling effect lasted for at least 20 days (5 passages), indicating good retention of internalized TAT-CPNPs in MSC^Cxcr3^. Unlabeled MSC^Cxcr3^ showed a similar PA signal intensity to double distilled water (DW) under 1064 nm laser excitation (Fig. 6b, lane 2 vs lane 1). Upon TAT-CPNPs uptake, the PA signal intensity of MSC^Cxcr3^ enhanced 17.61-fold (Fig. 6b, lane 3 vs lane 2). Based on the PA amplitude-concentration linear fit equation in Fig. 2f, the uptake rate of TAT-CPNPs was estimated to be approximately 39.3% and the amount of initially internalized TAT-CPNPs was approximately 23.60 pg/cell. Even after 10 days and 20 days, the cellular PA signal intensity was still 9.6-fold and 6.97-fold higher than that of unlabeled ones (Fig. 6b, lane 4 and 5 vs lane 2) with remaining amounts of internalized TAT-CPNPs of approximately 13.4 and 9.9 pg/cell, respectively. These results indicated the great potential of TAT-CPNPs as long-term MSC tracers, which conformed to the requirement for long-lasting monitoring in vivo.

To evaluate the PA imaging performance of TAT-CPNPs labeled MSC^Cxcr3^ located within biological tissues, MSC^Cxcr3^ under various thicknesses of chicken breast tissue were subjected to PA imaging. As shown in Fig. 6c, the PA signal of either unlabeled MSC^Cxcr3^ under 1064 nm laser excitation or TAT-CPNPs labeled MSC^Cxcr3^ under 800 nm laser excitation was barely distinguishable from background noise regardless of the thickness of the covering chicken breast tissues. After culturing with TAT-CPNPs, TAT-CPNPs labeled MSC^Cxcr3^ showed a significant PA signal under 1064 nm laser excitation and the corresponding PA signal was still detectable even when covered by 12 mm of chicken breast tissue. Comparatively, the penetration depth of conventional optical imaging methods, such as confocal microscopy and two-photon microscopy are restricted to < 500 µm. Therefore, these results demonstrated the potential of TAT-CPNPs-assisted NIR-II PA imaging for deep-tissue located MSCs.

### Serial NIR-II PA imaging for assessing the targeting migratory ability of MSCs

MSC homing is defined as the arrest of MSCs within the vasculature of a tissue followed by their transmigration across the endothelium [7]. Identification of the relative position of MSCs to blood vessels reveals the status of MSC homing more specifically compared to simply addressing the concentration of MSCs within target sites. A few current studies integrated photoacoustic imaging of blood vessel and MSCs [22, 33]. However, in terms of the delivery route, while systemic delivery by intravenous injection is the major delivery route for most systemic diseases, MSCs were mainly delivered locally (e.g., superficial application, subcutaneous or orthotopic administration, etc.) in related research. In order to reveal the small amount of MSCs delivered by intravenous injection, unveil the possible difference between normal and chemokine receptor-gene modified MSCs and precisely measure the relative position of MSCs to blood vessels, an imaging strategy with high detection sensitivity is needed. To choose among imaging strategies for longitudinal MSC imaging, a comparison of the cell detection threshold and other essential characteristics was conducted among several imaging strategies. Although nuclear medicine showed nanomolar sensitivity, it was excluded due to the short half-life of radioisotopes and repeated radiation exposure during longitudinal tracking. Two-photon microscopy with subcellular resolution was also excluded due to a field-of-view of a diameter < 1 mm [34]. For magnetic resonance imaging (MRI) using 4.7 T magnet, the lower limit of detection was approximately 500 cells/mm^3^ when cells were labeled with iron at 25 µg/mL [35]. For PA imaging, the reported detection limit for ultrasound-guided PA imaging was < 10 cells/mm^3^ with a customized field of view and no radiation [33]. In particular, OR-PAM scans a tightly focused laser beam in the optical ballistic regime and provides micrometer-scale resolution. OR-PAM was promising for fulfilling the requirement of detection sensitivity and was thus chosen for measurement of targeting migration of MSCs in vivo.

The in vivo targeting migration ability of MSCs towards inflamed ears of the CHS model was evaluated using longitudinal NIR-II PA imaging. Before injection of MSCs, PA imaging was first performed at a wavelength of 532 nm to image the ear vasculature and at 1064 nm to acquire the background signal [18]. Then, PA imaging under 1064 nm excitation was performed at 15 min, 3 h and 7 h after intravenous delivery of TAT-CPNPs labeled MSC^eGFP^ or MSC^Cxcr3^, generating differential images by subtraction of the preinjection image from the postinjection image. The coregistration of PA images obtained at 532 nm and 1064 nm facilitated the assessment of the relative location of MSCs and vessels, which helped measure the targeting migration status of delivered MSCs.

**Fig. 7:**
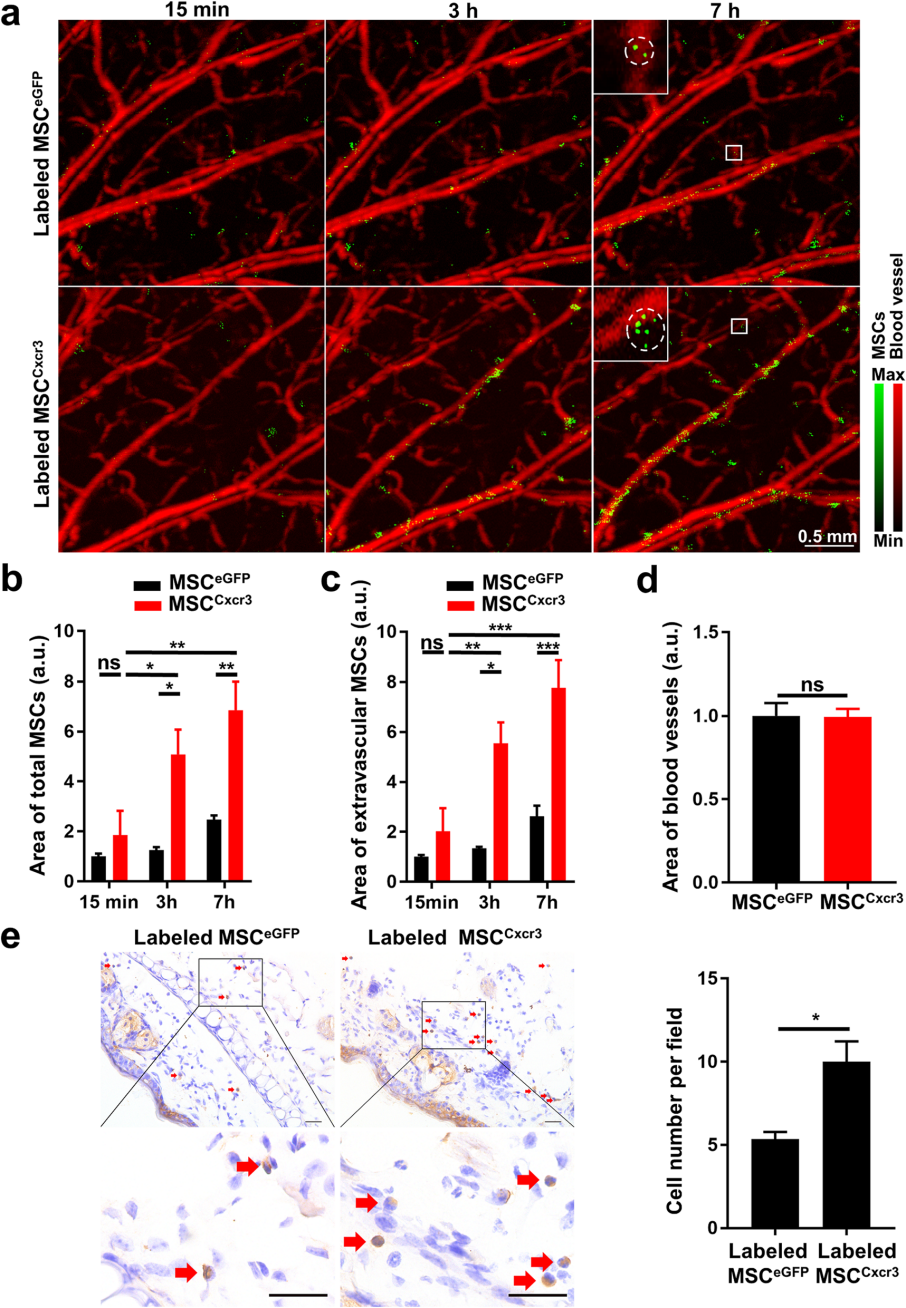
MSC^Cxcr3^ exhibited enhanced migration toward inflamed ears in vivo. **a** Representative PA MAP images showing more significant accumulation of TAT-CPNPs labeled MSC^Cxcr3^ than MSC^eGFP^ in the inflamed ears of CHS mice over time. PA images were captured at 15 min, 3 h or 7 h after mice receiving labeled MSC^eGFP^ or MSC^Cxcr3^ injections, respectively. The imaging procedure for each group was repeated on 3 mice and complemented in Fig. S4. White boxes correspond to magnified region in upper left. White dashed circles indicate TAT-CPNPs labeled MSC^eGFP^ or MSC^Cxcr3^. Scale bar, 0.5 mm. **b** Normalized area of total labeled MSC^eGFP^ or MSC^Cxcr3^ in PA images captured 15 min, 3 h and 7 h after labeled MSC^eGFP^ or MSC^Cxcr3^ injection (n = 3 per group). **c** Normalized area of extravascular labeled MSC^eGFP^ or MSC^Cxcr3^ in PA images captured 15 min, 3 h and 7 h after labeled MSC^eGFP^ or MSC^Cxcr3^ injection (n = 3 per group). **d** Normalized area of blood vessels of inflamed ears of CHS mice injected with MSC^eGFP^ or MSC^Cxcr3^ (n = 3 per group). **e** Histological examination verified the significantly increased presence of labeled MSC^Cxcr3^ compared with labeled MSC^eGFP^ in the inflamed ear of CHS mice by immunostaining of GFP. The red arrows in the magnified images denoted GFP-expressing MSCs. Scale bars, 25 µm. ns, no significance; *P < 0.05, **P < 0.01, ***P < 0.001; error bars, SEM

In control mice, the PA signal distribution profiles remained similar at all time points, suggesting a negligible effect on PA images caused by repeated l064 nm laser excitation and MSC injection (Additional file 1: Fig. S3a). 15 min after the injection of TAT-CPNPs labeled MSC^eGFP^ or MSC^Cxcr3^, some high-intensity PA signal speckles emerged in proximity to blood vessels (Fig. 7a, and Additional file 1: Fig. S4). At 3 h and 7 h after injection, the area of high-intensity PA signal speckles increased gradually over time (Fig. 7a, b), which was not observed in non-inflamed ears (Additional file 1: Fig. S3b), and the high-intensity PA signal speckles appeared from intravascular to extravascular area (Fig. 7a and c), supporting that more injected MSC^Cxcr3^ achieved targeting migration and extravasation to inflamed ears compared to MSC^eGFP^ (Fig. 7a-d, Additional file 1: Fig. S4). The targeting migratory capacity of MSC^eGFP^ and MSC^Cxcr3^ was further confirmed by immunohistochemistry (IHC) staining of green fluorescence protein (GFP). As shown in Fig. 7e, there were more GFP^+^ cells in the inflamed ears of CHS mice receiving labeled MSC^Cxcr3^ injection than in those receiving MSC^eGFP^ injection, which was in accordance with the trend suggested by PA imaging.

Notably, the layer-by-layer and xz-projected PA images of an inflamed ear of a CHS mouse showed detection of both blood vessels and MSCs through a thickness of more than 420 µm of mouse ear (Additional file 1: Fig. S5, S6), suggesting that our imaging strategy conformed to the imaging depth requirement for inflamed ears with a mean thickness of 0.42 mm (Fig. 4b). Compared with other microscopy imaging modalities, such as confocal laser scanning microscopy and two-photon microscopy, the imaging depth of confocal microscopy is limited to 100 to 200 µm [36]. Using near-infrared laser excitation, two-photon microscopy is capable of imaging ~ 450 µm below the surface of living tissues if there are few fluorophore molecules outside the focal plane; also, the cost remains generally high [37, 38]. In addition, fine resolution and signal-background ratio were well maintained within tissue using OR-PAM. As shown in Additional file 1: Fig. S5b, under 532 nm excitation, blood vessels with a diameter of 18.77 µm were clearly visualized at a depth of 308 µm and an average signal-to-background ratio (SBR) of 13.01 dB. Under 1064 nm excitation, TAT-CPNPs labeled MSC^Cxcr3^ at the same depth was clearly identified with an average SBR of 13.40 dB. Accordingly, TAT-CPNPs-assisted OR-PAM imaging was able to provide essential information about the location of MSCs relative to blood vessels in addition to their quantification.

In addition, we also explored the potential of applying TAT-CPNPs-assisted NIR-II PA imaging of MSCs in a deeper inflammation animal model. Since Cxcl10 has also been reported to be up-regulated in the inflamed limbs of a rheumatoid arthritis (RA) model [39], a preliminary investigation was carried out to explore imaging exogenously delivered MSC^eGFP^ and MSC^Cxcr3^ in inflamed hindlimbs of RA mouse model. As shown in Additional file 1: Fig. S7, TAT-CPNPs assisted NIR-II PA imaging successfully showed the transplanted MSCs at a depth of 1.0 mm and also discerned the enhanced targeting migratory ability attributed to Cxcr3 modification.

Collectively, these results suggested that TAT-CPNPs-assisted NIR-II PA imaging was able to noninvasively track the targeting migration of intravenously transplanted MSCs in vivo and could be helpful for selecting molecular targets for improving the homing ability of MSCs.

## Conclusion

Current studies on PA imaging of MSCs mostly transplanted MSCs by subcutaneous or orthotopic injection. We first investigated NIR-II OR-PAM imaging of intravenously delivered MSCs to simulate the clinical delivery route of MSCs for the treatment of systemic disease. In this study, we described a facile strategy to evaluate the effect of chemokine-receptor gene modification on the migratory ability of MSCs by using intravital NIR-II OR-PAM imaging. The prepared TAT-CPNPs exhibited great biocompatibility and strong NIR-II absorbance, enabling efficient, long-lasting, and deeply penetrating NIR-II PA imaging of MSCs. Combining the advantages of high resolution and label-free vessel imaging of OR-PAM imaging, information about the distribution of MSCs with high SBR as well as their relative position to blood vessels was acquired. Furthermore, more TAT-CPNPs labeled MSC^Cxcr3^ gradually migrated to CHS inflamed lesions and achieved extravasation at 3 h and 7 h after intravenous administration compared to MSC^eGFP^, indicating enhanced targeting migratory capacity attributed to Cxcr3 gene modification. Hence, this work illustrates the good performance of TAT-CPNPs-assisted OR-PAM imaging in evaluating the targeting migratory capacity of MSCs to disease sites and unveils the potential of Cxcr3 modification in therapeutic MSC delivery to inflammation with high production of Cxcl10. Our labeling and imaging strategy may facilitate studies on screening genes for enhancing targeting migration, offering a guidance to improve the therapeutic efficacy of MSCs. In addition to skin diseases, future research can also focus on assessing the targeting migratory ability of modified MSCs in deeper located diseases sites, especially those with solid evidence of MSC therapy.

## Methods

### Materials

Toluene, chloroform, tetrahydrofuran (THF), acetone and sucrose were purchased from Guangzhou Chemical Reagent Factory (Guangzhou, China). Olive oil (O108686) was provided by Aladdin (Shanghai, China). 2,5-Bis(2-octyldodecyl)-3,6-bis(5-(trimethylstannyl)thiophen-2-yl)-2,5-dihydropyrrolo[3,4-c]pyrrole-1,4-dione (DPP) and 4,8-dibromo-6-(2-ethylhexyl)-[1,2,5]thiadiazolo[3,4-f]benzotriazole (TBZ) were purchased from Derthon Optoelectronic Materials Science Technology Co., Ltd. (Guangdong, China). Tris(dibenzylideneacetone)dipalladium(0), tri(*o*-tolyl)phosphine and DNFB (D1529) were supplied from Merck Millipore (Darmstadt, Germany). DSPE-PEG_2000,_ (molecular weight: 2805.5) and DSPE-PEG_2000_-MAL were purchased from Xi’an ruixi Biological Technology Co., Ltd. (Xi’an, China). Transactivator of transcription (TAT) peptide (296229) was purchased from GL Biochem, Ltd. (Shanghai, China). Dulbecco’s Modified Eagle Medium (DMEM), fetal bovine serum (FBS, 10099141), 0.25% trypsin-ethylenediaminetetraacetic acid (25200056), TRIzol™ Reagent and RevertAid First Strand cDNA Synthesis Kit (K1622) were purchased from Invitrogen (Carlsbad, CA, USA). Lenti-Pac HIV Expression Packaging Kit (LT001) and polybrene (LT010-S) were purchased from IGeneBio (Guangzhou, China). Radioimmunoprecipitation (RIPA) lysis buffer (P0013B) was purchased from Beyotime Biotech Inc. (Shanghai, China). Bovine serum albumin (BSA, A8020) was purchased from Beijing Solarbio Science & Technology Co., Ltd. (Beijing, China). Transfer membrane, goat polyclonal anti-rabbit horseradish peroxidase (HRP) conjugated antibody (ab205718), rabbit polyclonal antibody against GFP (ab290), 3,3'-Diaminobenzidine (DAB) substrate kit (ab64238) were purchased from Abcam (Cambridge, MA, USA). Tris buffered saline with tween (TBST), PBS, crystal violet, H&E staining kit (G1005) and 4% paraformaldehyde solution (PFA, G1101) were purchased from Wuhan Servicebio Technology Co., Ltd. (Wuhan, China). Rabbit polyclonal antibody against Cxcr3 (NB100-56404), recombinant Cxcl10 protein (466-CR) were purchased from R&D systems (Minneapolis, MN, USA). Meilunbio® fg super sensitive electrochemiluminescence luminescence reagent (MA0186) was purchased from Dalian Meilun Biotech Co., Ltd. (Dalian, China). CCK-8 reagent (KGA317) was purchased from KeyGen Biotech Co., Ltd. (Nanjing, China). TB Green Premix Ex Taq II (Tli RNase H Plus) (RR820A) was purchased from Takara Bio Inc. (Otsu, Japan). All other chemicals were used as received.

### Preparation of TAT-CPNPs

Conjugated polymer PTD was synthesized by alternative copolymerization between TBZ and thiophene-substituted DPP monomers via Stille coupling reaction. For imaging in vitro and in vivo, PTD molecules were processed into water dispersible conjugated polymer nanoparticles (CPNPs) via a nanoprecipitation method. The process procedure is described briefly as follows. PTD (1 mg), DSPE-PEG_2000_-MAL (1 mg) and DSPE-PEG_2000_ (1 mg) were dissolved in THF (2 mL) and dispersed in DW under ultrasonification (Ultrasonic Homogenizer SCIENTZ-IID, Ningbo Scientz Biotechnology Co., Ltd., Ningbo, China). After THF evaporation, the mixture was filtered through 450-nm-pore polyether sulfone filters. To prepare TAT-CPNPs, TAT peptide (3.3 mg) was added to the filtered mixture and stirred for 12 h. The mixture was dialyzed against DW for 24 h and concentrated to about 1 mg/mL with Amicon® Ultra Centrifugal Filters (UFC9010, Merck Millipore, Germany).

### Characterization of TAT-CPNPs

The size distribution of TAT-CPNPs was determined by dynamic light scattering with Zetasizer Nano ZS system (Malvern Instruments, Malvern, UK). Transmission electron microscopy (TEM) images of TAT-CPNPs were taken with FEI TECNAI G2 20 transmission electron microscope (FEI, Hillsboro, USA) at 200 kV. Ultraviolet–visible-near infrared spectra of TAT-CPNPs and absorbance of TAT was measured with an ultraviolet–visible-near infrared spectrophotometer (PerkinElmer Inc., Waltham, USA). PA signal intensities of TAT-CPNPs at 10, 20, 100, 200, 2000 µg/mL and PA signal intensity of MSCs cultured with CPNPs and TAT-CPNPs for different time (1 h, 16 h, 24 h) were measured with a custom-built PACT at 10 mJ/cm^2^ as previously described in literature. Photostability was assessed by quantification of PA signal intensity of TAT-CPNPs during continuous 2000 pulses of 1064 nm laser irradiation.

### Quantification of TAT linked to CPNPs

To quantify the TAT being linked to CPNPs, the free TAT concentration in CPNPs suspension was measured, and the linked amount of TAT was calculated according to the free TAT concentration. The free TAT concentration in CPNPs suspension was measured as follows. After preparing CPNPs with PTD (0.36 mg), DSPE-PEG_2000_-MAL (0.36 mg) and DSPE-PEG_2000_ (0.36 mg), TAT (1.19 mg) was added to the CPNPs. After stirring for 12 h, the mixture was collected and freeze-dried. 50 µL of DW was added to freeze-dried TAT-CPNPs to obtain the aqueous solution containing free TAT. Using bicinchoninic acid (BCA) protein assay kit (Beyotime Biotechnology, Shanghai, China), the free TAT in the solution was estimated to be 1.04 mg based on the absorbance-TAT concentration linear fit equation in Additional file 1: Fig. S1. The TAT linked to CPNPs was calculated to be 0.16 mg (0.05 µmol). Based on the 0.36 mg of DSPE-PEG_2000_-MAL at the feed, the available maleimide was 0.12 µmol. Therefore, 45% of maleimide on the surface of TAT-CPNPs was linked to TAT [28].

### Lentiviral vectors and lentivirus production

Entry vectors were generated according to previous literature. The lentiviral expression vector with the coding sequence of Cxcr3 (pLV/puro-EF1a-CXCR3-IRES-EGFP) was generated using a lentiviral vector that expressed eGFP (pLV/ puro-EF1a-EGFP).

For lentivirus production, HEK293T cells were transiently cotransfected with lentiviral expression vector (pLV/puro-EF1a-EGFP or pLV/puro-EF1a-CXCR3-IRES-EGFP) together with packaging plasmids from the Lenti-Pac HIV Expression Packaging Kit. The supernatant was collected 36 h and 72 h after transfection and then filtered through 0.45-µm-pore polyether sulfone filters. The lentiviral particles were concentrated by ultracentrifugation (2 h at 50,000×*g*) and resuspended in serum-free medium (SFM).

### Cell culture

MSCs were isolated from human bone marrow samples obtained from healthy human donors with informed consent as described in previous literature [8]. MSCs were routinely cultured in DMEM complete medium (CM) containing 1 g/L glucose and 10% FBS. HEK293T were routinely cultured in DMEM CM containing 4.5 g/L glucose and 10% FBS. Cells were cultured at 37 ℃ and 5% carbon dioxide (CO_2_) in a CO_2_ incubator.

MSCs stably overexpressing Cxcr3 (MSC^Cxcr3^), and its matched control MSC^eGFP^ were established using lentiviral transduction. After reaching a confluence of approximately 60%, MSCs were cultured in CM containing lentivirus and 8 µg/mL polybrene for 12 h. The eGFP-expressing cells were observed under a fluorescence microscope (Olympus IX73 microscope, Olympus, Inc., Hamburg, Germany) or subjected to flow cytometry (CytoFLEX, Beckman Coulter, Inc., CA, USA) to assess the transduction efficiency.

### In vitro cytotoxicity assay

MSCs were resuspended in CM at 1.5 × 10^5^ cells/mL. In a 96-well plate, 100 µL of cell suspension was added to each well. After incubation at 37 ℃ and 5% CO_2_ for 24 h, MSCs were cultured with serum-free DMEM medium containing CPNPs or TAT-CPNPs at different concentrations (0, 3.25, 7.5, 15, 30, 60, 120 µg/mL). After 12 h, cell viability was measured with CCK-8 assay according to the manufacturer’s protocol.

### Mice

BALB/c male mice (6 weeks) and DBA/2J male mice (8 weeks) were purchased from Beijing Vital River Laboratory Animal Technology Co., Ltd. All animal studies were carried out in accordance with the guidelines of the Institutional Animal Care and Use Committee of Shenzhen Institutes of Advanced Technology, Chinese Academy of Sciences.

### In vivo toxicity assay

100 µL of PBS containing TAT-CPNPs at 0 or 500 µg/mL was injected intravenously into mice. After 14 days, major organs (heart, liver, spleen, lungs, and kidneys) were collected from the treated mice for hematoxylin and eosin (H&E) staining. Serum was collected for biochemistry analysis with a Chemray-240 automated chemistry analyzer (Rayto Life and Analytical Sciences Co., Ltd., Guangdong, China). Parameters including AST, ALT, TBIL, Cr and BUN for liver and kidney function assessment were analyzed.

### Establishment of the CHS model and ear thickness measurement

The CHS model was established as described in literature [40]. Acetone and olive oil at a ratio of 4:1 was prepared to dilute DNFB. 0.5% DNFB was applied to shaved mice back for sensitization. After 5 days, 0.2% DNFB was applied to the right ears in inflamed group, vehicle was applied to left ears in control group. The ear thickness of both ears was measured before and 24 h and 72 h after 0.2% DNFB or vehicle application.

### Analysis of gene expression

Gene expression was analyzed by real-time quantitative reverse transcription polymerase chain reaction (qRT-PCR) and western blotting.

For mRNA analysis, total RNA was extracted with TRIzol™ Reagent according to manufacturer’s protocol. Reverse transcription was carried out with a RevertAid First Strand cDNA Synthesis Kit according to the manufacturer’s protocol. qRT-PCR was carried out with TB Green Premix Ex Taq II (Tli RNase H Plus) using qTower 3 (Analytik Jena AG, Thuringia, Germany). qRT-PCR was conducted in triplicate for each sample. Primers for qRT-PCR were synthesized by Sangon Biotech Co., Ltd. (Shanghai, China) and are listed as follows. Mouse Cxcr3, forward, 5′-TCAGCCAACTACGATCAGCG-3′; reverse, 5′-TAGCTGCAGTACACGCAGAG-3′. Human GAPDH, forward, 5′-GGAGCGAGATCCCTCCAAAAT-3′; reverse, 5′-GGCTGTTGTCATACTTCTCATGG-3′. Mouse Cxcl10, forward, 5′-CCAAGTGCTGCCGTCATTTTC-3′; reverse, 5′-GGCTCGCAGGGATGATTTCAA-3′. Mouse GAPDH, forward, 5′-AGGTCGGTGTGAACGGATTTG-3′; reverse, 5′-TGTAGACCATGTAGTTGAGGTCA-3′.

For western blotting, MSCs were lysed with RIPA lysis buffer. Proteins were separated with sodium dodecyl-sulfate polyacrylamide gel electrophoresis and detected with specific antibodies. Blots were detected with the Meilunbio® fg supersensitive electrochemiluminescence luminescence reagent in a BLT GelView 6000 Pro (Biolight Biotechnology Co., Ltd., Guangzhou, China) system.

### Transwell migration assay

Migration was assessed with a 24-well transwell chamber system (353097, Corning Costar, Cambridge, MA, USA) with 8 µm pores. To evaluate the chemotactic response to Cxcl10, MSC^eGFP^ and MSC^Cxcr3^ were resuspended in serum-free DMEM medium at 1 × 10^5^ cells/mL. Then, 150 µL of cell suspension was added to each upper chamber insert. The lower chamber contained CM with or without murine Cxcl10 (100 ng/mL). To investigate the effect of TAT-CPNPs on chemotactic migration, MSC^Cxcr3^ were resuspended in serum-free DMEM medium containing 15 µg/mL of TAT-CPNPs or an equivalent amount of vehicle (PBS) at 1 × 10^5^ cells/mL, and 200 µL of cell suspension was added to each upper chamber insert. The lower chamber contained CM with murine Cxcl10 (100 ng/mL). For both experiments, cells were allowed to migrate to the lower chamber at 37 ℃ and 5% CO_2_ for 24 h. After that, the insert was fixed with ice-cold methanol, the upper surface was scraped clean and the lower surface was stained with 0.1% crystal violet. Images of migrated cells were captured with an Olympus IX73 microscope (Olympus, Inc., Hamburg, Germany). The number of migrated cells in each chamber was determined from images of 5 nonrepeating random fields.

### Labeling of MSCs

When MSC^Cxcr3^ reached a confluence of 70%, cells were cultured with SFM containing 15 µg/mL of TAT-CPNPs for 16 h. To compare the uptake of CPNPs and TAT-CPNPs, MSCs were cultured with SFM containing 15 µg/mL of CPNPs or TAT-CPNPs for different amount of time (1 h, 16 h or 24 h). After washing with PBS for 3 times, cells were trypsinized either for subsequent PA imaging or subculture.

### PA imaging of MSCs in vitro

After incubating with TAT-CPNPs, the culture plate was washed with PBS and the culture medium was changed to normal complete medium. Labeled-MSC^Cxcr3^ were cultured and passaged as usual for 0 day, 10 days and 20 days, respectively. Then, MSC^Cxcr3^ resuspended in PBS at 2 × 10^6^ cells/mL were loaded into a tube phantom and PA imaging was performed with the PACT system. The amounts of NPs initially internalized by MSCs and remained in cells after different time were quantified based on the PA amplitude-concentration linear fit equation in Fig. 2f.

To compare the penetration performance of PA imaging at first and second near-infrared windows, the cell-containing tube phantoms were placed underneath approximately 5 mm, 8 mm or 12 mm of chicken breast tissue before PA imaging. Both ultrasonic and PA signals were coupled and detected by a 15 MHz transducer. PA imaging was performed at 10 mJ/cm^2^ under 800 nm or 1064 nm laser excitation [41].

### In vivo PA imaging of CHS model

To investigate the recruitment of MSCs to inflamed ears in vivo, a custom-built OR-PAM system was employed under 532 nm and 1064 nm laser excitation. 24 h after application of 0.2% DNFB, CHS mice were randomly assigned to 2 groups. Mice were intravenously injected with TAT-CPNPs labeled MSC^eGFP^ (2 × 10^6^ cells, n = 3) and TAT-CPNPs labeled MSC^Cxcr3^ (2 × 10^6^ cells, n = 3), respectively. Imaging of the inflamed ears of another 3 mice injected with PBS, unlabeled MSC^eGFP^ or unlabeled MSC^Cxcr3^ group and imaging of the non-inflamed ear of another mouse injected with labeled MSC^Cxcr3^ were included as the control group. Each mouse was anesthetized with 2% isoflurane in oxygen and then sedated in prone position. During imaging, the mouse body temperature was maintained at 37 °C using a temperature-controlled heating pad (RWD Life Science, Shenzhen, China). PA images of inflamed ears were captured at a wavelength of 532 nm first to reveal the vasculature. Then, PA images at a wavelength of 1064 nm were captured before, 15 min, 3 h and 7 h after injection of MSC^eGFP^ or MSC^Cxcr3^. During the experiment, the fluence of each PA imaging on tissue surface of each mouse was kept at approximately 15 mJ/cm^2^ at a wavelength of 532 nm and 64 mJ/cm^2^ at a wavelength of 1064 nm by monitoring using a power sensor (S121C, Thorlabs, New Jersey, USA). Fluences applied at both wavelengths were well below the maximum permissible exposure standard (20 mJ/cm^2^ at a wavelength of 532 nm and 100 mJ/cm^2^ at a wavelength of 1064 nm) allowed by the American National Standard Institute (ANSI Z136. 1-2014: American National Standard for Safe Use of Lasers).

The raw 3D volume data for each animal at each time point acquired by PA imaging were processed using Matlab (R2021a) to show the maximum amplitude projection (MAP) image. The detailed imaging processing algorithm was according to previous literature from our group. For each ear, images captured at a wavelength of 1064 nm were aligned to image captured at 532 nm using the TurboReg plugin in Image J (Software version 1.8.0_112) before further processing. After alignment, the PA signal of TAT-CPNPs labeled MSCs was extracted by subtracting the pre- from postinjection PA images under 1064 nm using Matlab (R2021a). Finally, the subtracted PA image of each time point was merged with image captured at 532 nm to reveal the position of MSCs relative to blood vessels. Quantification analysis of PA images captured in vivo was performed using Image J. Layer-by-layer images and xz projected images were processed using Matlab and analyzed using Image J.

### Establishment and in vivo PA imaging of RA mouse model

The RA mouse model was established following the booster immunization protocol from Chondrex, Inc,.(CA, USA). Briefly, an emulsion of collagen and complete Freund’s adjuvant with a final concentration of 0.5 mg/mL of M. tuberculosis was prepared with a homogenizer (T 10 basic ULTRA-TURRAX, IKA, Staufen, Germany). 100 µl of emulsion was subcutaneously injected into the base of the tail of DBA/2J mice. 21 days later, a booster injection was performed with an emulsion of collagen and Incomplete Freund’s Adjuvant. Arthritis was scored following the scoring system reported by D. Brand et.al [42]. Approximately 15 days after the booster immunization, in vivo PA/US imaging was performed on the hindlimb with a severity score of 3–4 using the PACT system. Briefly, mice were sedated by continuous isoflurane inhalation. PA/US imaging was performed before and 15 min, 2 h and 4 h after injection of TAT-CPNPs labeled MSC^eGFP^ or MSC^Cxcr3^ under 1064 nm laser excitation. B-scan images of PA imaging were processed by subtracting the preinjection images from the postinjection images using Matlab. Subtraction images of PA imaging were overlaid with US images of related postinjection time points.

### Histological staining

IHC staining was performed to detect MSC^eGFP^ and MSC^Cxcr3^ in inflamed ears. After PA imaging, the ears were collected, fixed in 4% PFA for 12 h and dehydrated in 30% sucrose for 72 h to prepare cryosections. Sections were blocked with goat serum for 1 h at room temperature (RT) and then incubated with rabbit anti-GFP antibody (1:500 dilution) at 4 ℃ overnight, followed by incubation with goat anti-rabbit HRP conjugated antibody (0.1 µg/mL) for 1 h at RT. After washing with PBS, DAB was applied and sections were counterstained with hematoxylin. Photographs were captured with a KEYENCE BZ-X800 Microscope (Keyence Corporation, Osaka, Japan). Quantification of GFP positive cells in each mouse ear was from measurement of 5 non-repeating random fields.

### Statistical analysis

Data are expressed as means ± SEM and were analyzed by *t* tests and one-way analysis of variance (ANOVA) with Tukey’s posttest using Prism 7 software (GraphPad Software, Inc., CA, USA). Values of *P* < 0.05 were considered statistically significant.

## Supplementary Information

**Additional file 1.** Additional figures.

## Data Availability

Not applicable.

## Abbreviations

MSCs: Mesenchymal stem cells
NIR-II: Second near-infrared
TAT-CPNPs: Penetrating peptide-decorated conjugated polymer nanoparticles
Cxcl10: Chemokine (C-X-C motif) ligand 10
Cxcr3: Chemokine (C-X-C motif) receptor 3
PACT: PA computed tomography
PAM: Photoacoustic microscopy
OR-PAM: Optical-resolution photoacoustic microscopy
PTD: Poly(thiadiazolobenzotriazole-alt-ahiophene-substituted diketopyrrolopyrrole
CHS: Contact hypersensitivity
DSPE-PEG2000: 1,2-Distearoyl-sn-glycero-3-phosphoethanolamine-N-[methoxy(polyethylene glycol)-2000]
MAL: Maleimide
DLS: Dynamic light scattering
CCK-8: Cell Counting Kit-8
H&E: Hematoxylin and eosin
ALT: Alanine transaminase
AST: Aspartate aminotransferase
TBIL: Total bilirubin
Cr: Creatinine
BUN: Blood urea nitrogen
PBS: Phosphate buffered saline
ACD: Allergic contact dermatitis
DNFB: 2,4-Dinitro-1-fluorobenzene
eGFP: Enhanced green fluorescent protein
DW: Distilled water
MRI: Magnetic resonance imaging
IHC: Immunohistochemistry
GFP: Green fluorescence protein
SBR: Signal-to-background ratio
RA: Rheumatoid arthritis
THF: Tetrahydrofuran
DPP: 2,5-Bis(2-octyldodecyl)-3,6-bis(5-(trimethylstannyl)thiophen-2-yl)-2,5-dihydropyrrolo[3,4-c]pyrrole-1,4-dione
TBZ: 4,8-Dibromo-6-(2-ethylhexyl)-[1,2,5]thiadiazolo[3,4-f]benzotriazole
TAT: Transactivator of transcription
DMEM: Dulbecco’s Modified Eagle Medium
FBS: Fetal bovine serum
TEM: Transmission electron microscopy
CM: Complete medium
BCA: Bicinchoninic acid
CO_2_: Carbon dioxide
qRT-PCR: Real-time quantitative reverse transcription polymerase chain reaction
MAP: Maximum amplitude projection
