# Supplementary material for: Unveiling the improved targeting migration of mesenchymal stem cells with CXC chemokine receptor 3-modification using intravital NIR-II photoacoustic imaging
Source: J Nanobiotechnology. 2022 Jun 28;20:307. doi: 10.1186/s12951-022-01513-7 (PMC9238014; doi:10.1186/s12951-022-01513-7)
Supplement: Supplementary file 1 — Additional file 1. Additional figures. [file 12951_2022_1513_MOESM1_ESM.docx]

*Supporting information*

**Unveiling the Improved Targeting Migration of Mesenchymal Stem Cells with CXC Chemokine Receptor 3-Modification Using Intravital NIR-II Photoacoustic Imaging**

Yuejun Lin ^1#^, Hui-chao Zhou ^1#^, Ningbo Chen ^2#^, Yaguang Ren ^2^, Rongkang Gao ^2^, Qiaojia Li ^1^, Yiwen Deng ^4^, Xuejiao Han ^5^, Xiaoran Zhang ^4^, Andy Peng Xiang ^4^, Bing Guo ^3*^, Chengbo Liu ^2*^, Jie Ren ^1*^

^1^ Department of Ultrasound, Laboratory of Novel Optoacoustic/Ultrasonic imaging, Key Laboratory of Liver Disease of Guangdong Province, The Third Affiliated Hospital, Sun Yat-sen University, Guangzhou, 510630, China.

^2^ Research Laboratory for Biomedical Optics and Molecular Imaging, Shenzhen Institutes of Advanced Technology, Chinese Academy of Sciences, Shenzhen 518055, China.

^3^ School of Science and Shenzhen Key Laboratory of Flexible Printed Electronics Technology, Harbin Institute of Technology, Shenzhen, 518055, China.

^4^ Center for Stem Cell Biology and Tissue Engineering, Key Laboratory for Stem Cells and Tissue Engineering, Ministry of Education, Sun Yat-sen University, Guangzhou, 510630, China.

^5^ Department of Medical Oncology, Harbin Medical University Cancer Hospital, Harbin Medical University, Harbin, 150081, China

^#^ These authors contributed equally to this work.

^*^Corresponding author: Dr. Jie Ren, Department of Ultrasound, Laboratory of Novel Optoacoustic/Ultrasonic imaging, Guangdong Key Laboratory of Liver Disease Research, The Third Affiliated Hospital, Sun Yat-sen University, Guangzhou, 510630, China. Email: renj@mail.sysu.edu.cn. Dr. Chengbo Liu, Research Laboratory for Biomedical Optics and Molecular Imaging, Shenzhen Institutes of Advanced Technology, Chinese Academy of Sciences, Shenzhen 518055, China. E-mail: cb.liu@siat.ac.cn. Dr. Bing Guo, School of Science and Shenzhen Key Laboratory of Flexible Printed Electronics Technology, Harbin Institute of Technology, Shenzhen, 518055, China. E-mail: guobing2020@hit.edu.cn.


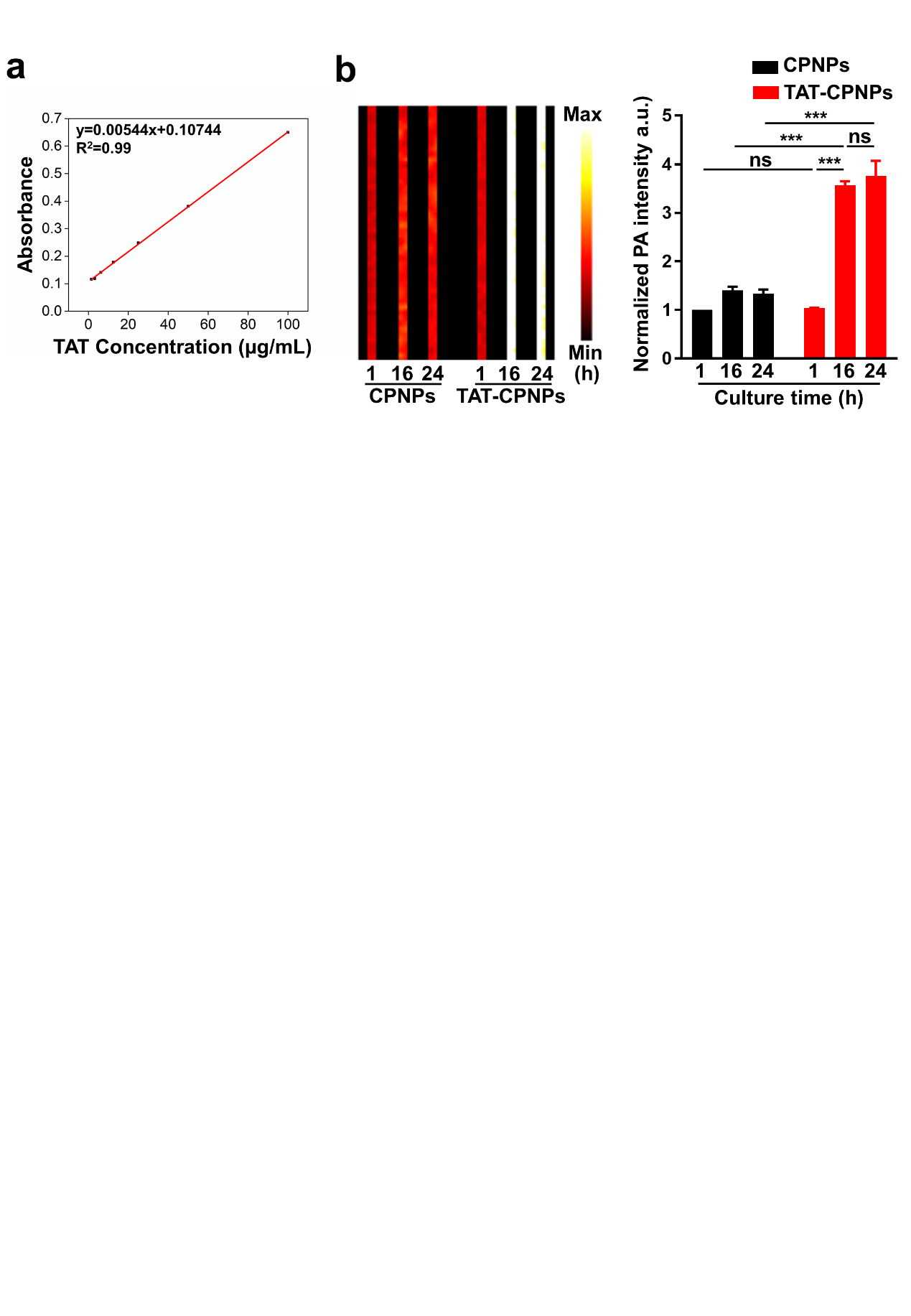


**Fig. S1** TAT peptide modification for CPNPs. **a** The linear fit of absorbance relative to TAT concentration. **b** Representative PA images and quantification analysis of MSCs that was cultured with CPNPs or TAT-CPNPs for different time (1 h, 16 h, or 24 h).


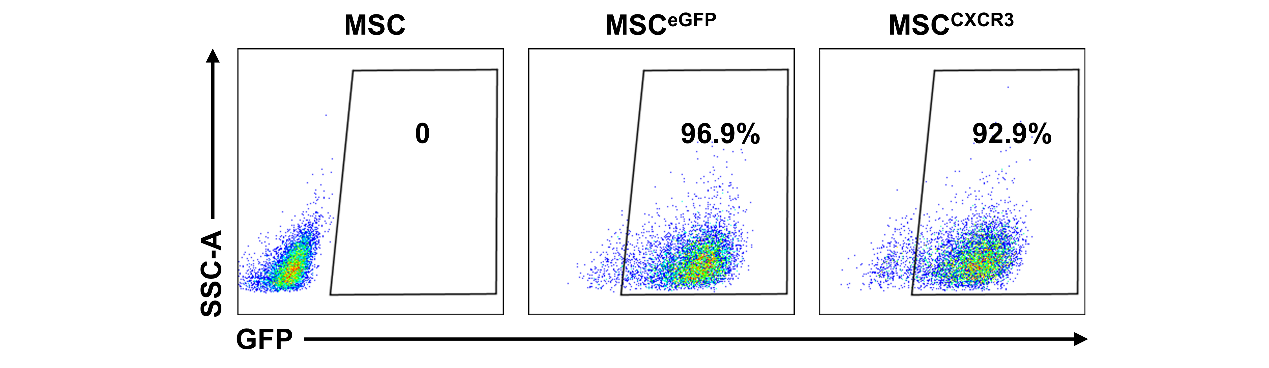


**Fig. S2** Representative flow cytometry histograms of GFP^+^ MSCs.


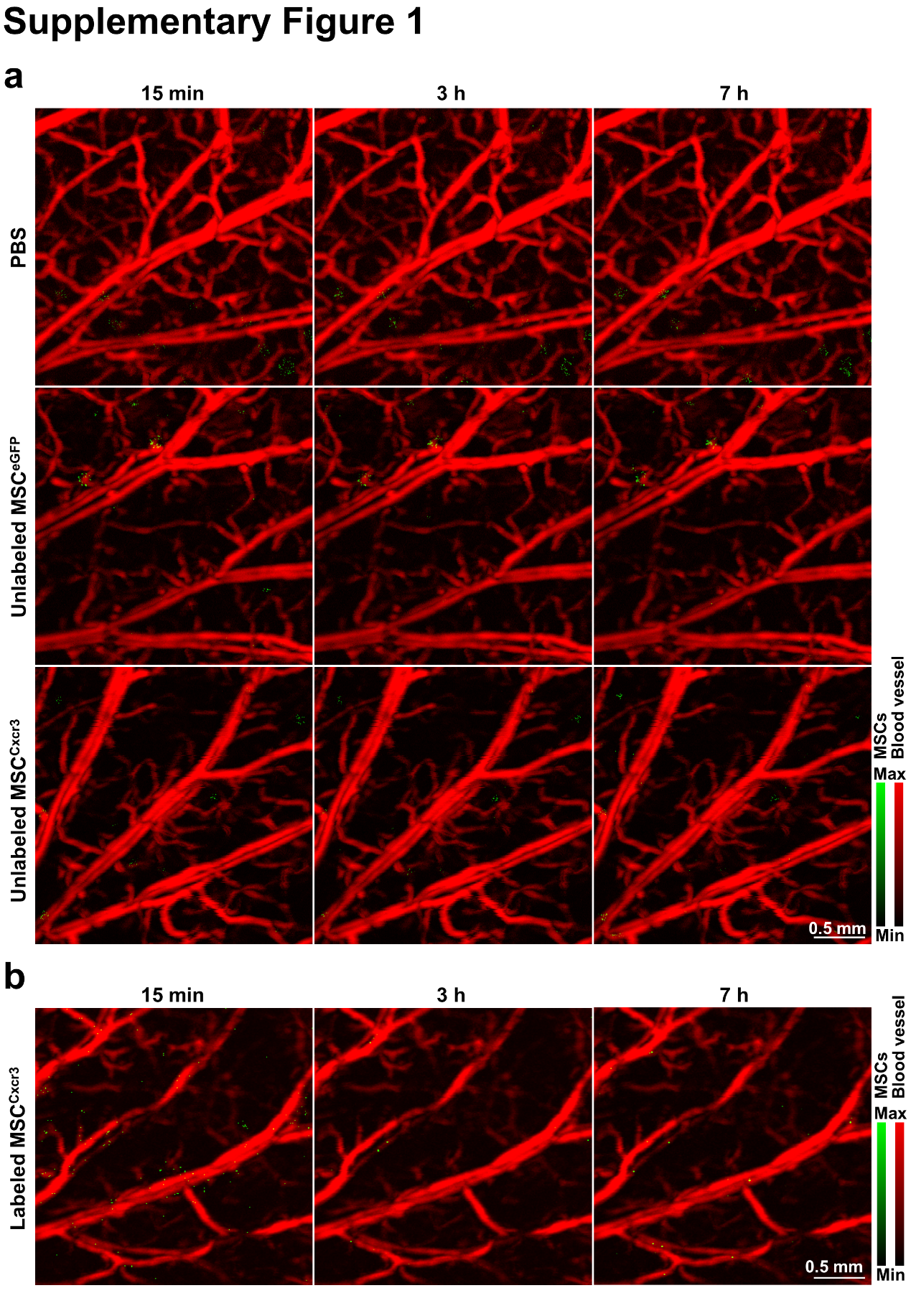


**Fig. S3** *In vivo* imaging of inflamed and non-inflamed ears of hypersensitivity mice. **a** Representative photoacoustic (PA) maximum amplitude projection (MAP) images of inflamed ears of contact hypersensitivity (CHS) mice receiving phosphate buffered saline (PBS), unlabeled MSC^eGFP^ or unlabeled MSC^Cxcr3^ injection. **b** Representative PA MAP images of non-inflamed ears of contact hypersensitivity (CHS) mice receiving TAT-CPNPs labeled MSC^Cxcr3^ injection. Representative PA MAP images were shown for 15 min, 3 h and 7 h after injections. Scale bar, 0.5 mm.


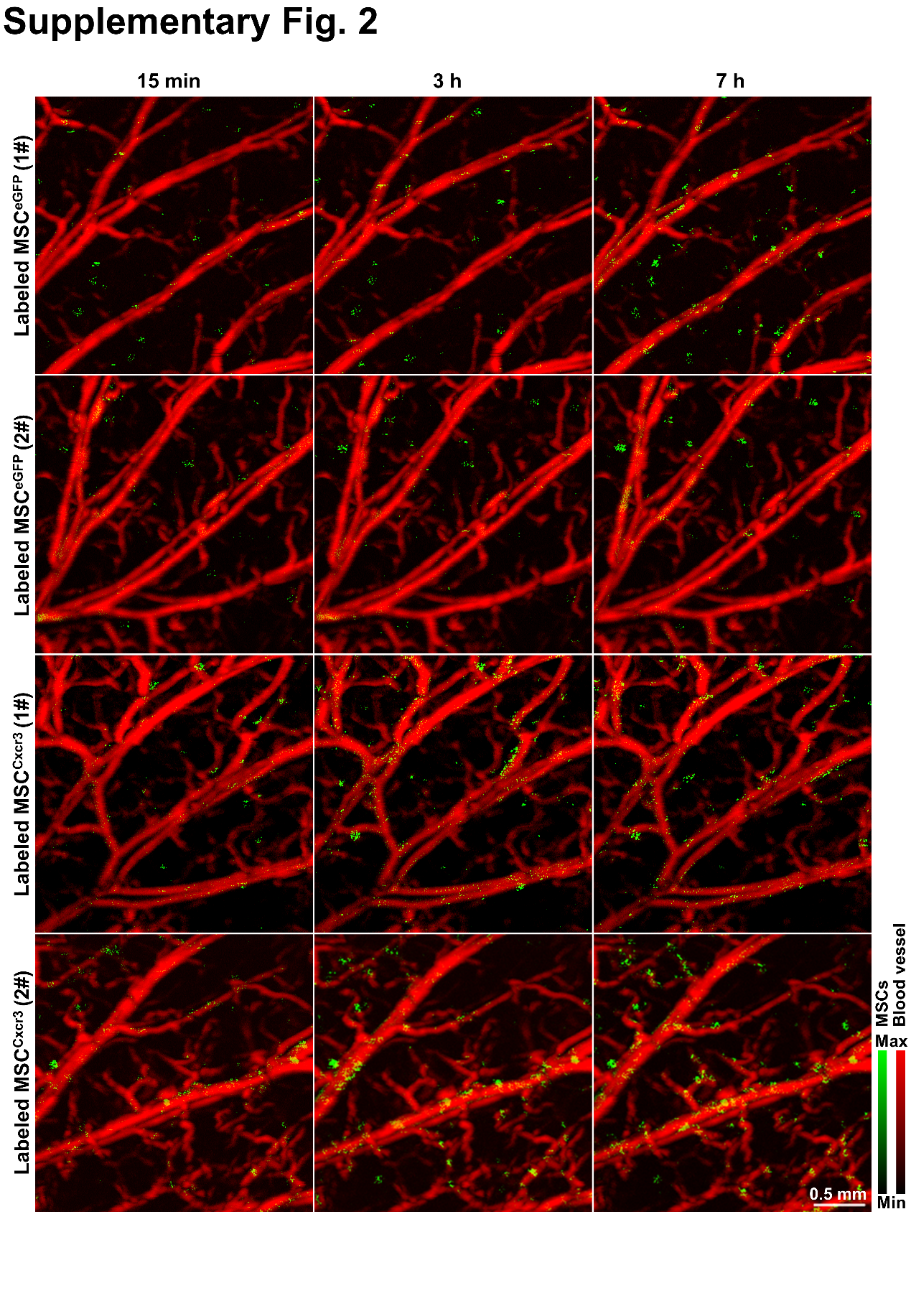


**Fig. S4** PA MAP images of inflamed ears of CHS mice receiving penetrating peptide-decorated biocompatible conjugated polymer nanoparticles (TAT-CPNPs) labeled MSC^eGFP^ or MSC^Cxcr3^ injection in relation to Figure 7a. The dynamic tracking of mesenchymal stem cells (MSCs) using PA imaging was performed for another 2 mice per group. Scale bar, 0.5 mm.


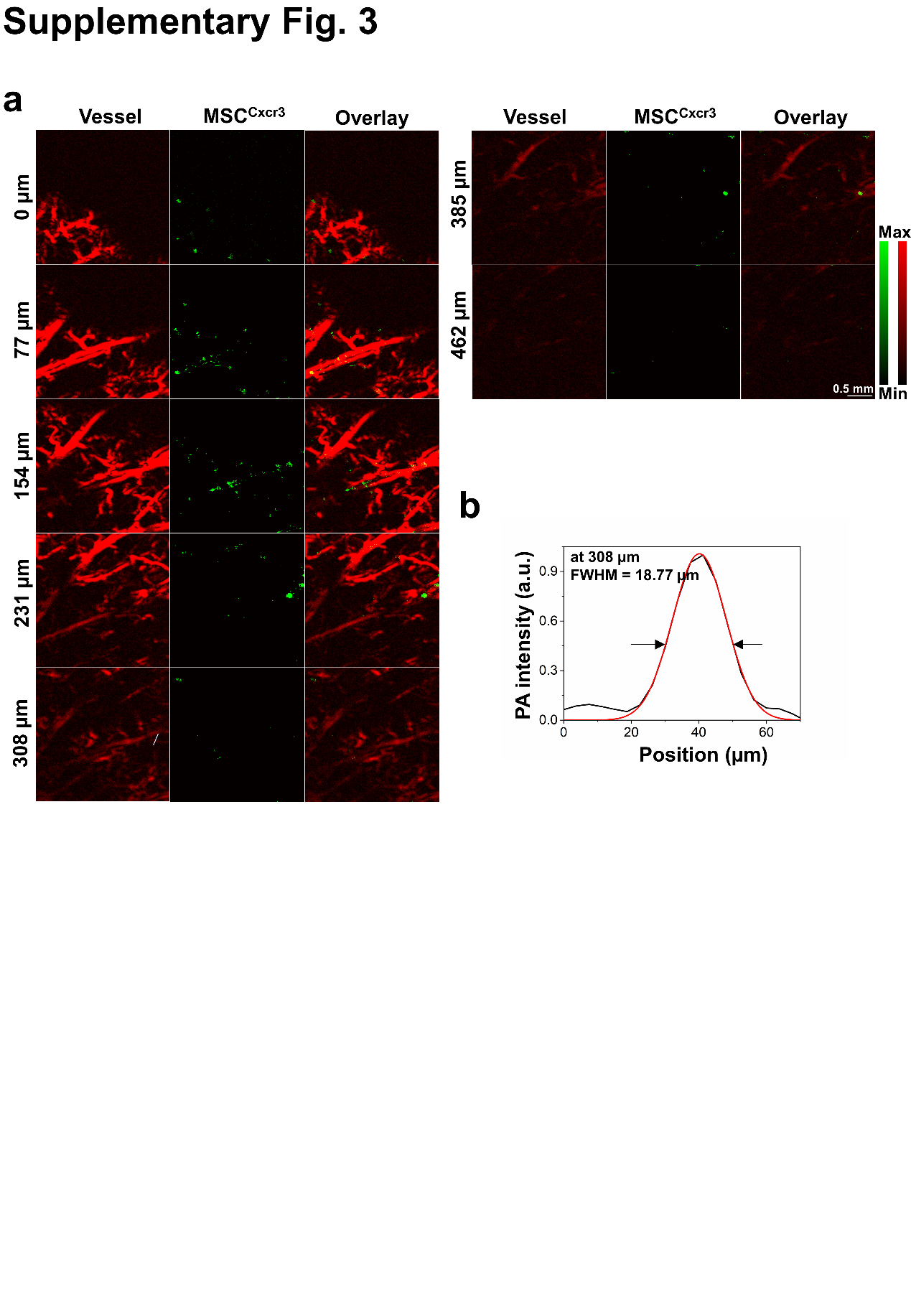


**Fig. S5** Layer-by-layer PA images of inflamed ear in relation to labeled MSC^Cxcr3^ (2#) in Fig. S3 and characterization of lateral resolutions of optical-resolution PA microscopy (OR-PAM) under 532 nm excitation. **a** PA images were reconstructed at different depth of inflamed ear of CHS mice after injection of TAT-CPNPs labeled MSC^Cxcr3^. White line indicated region for resolution measurement. **b** Lateral resolutions of OR-PAM under 532 nm excitation measured at 308 µm below the tissue surface. The cross-section profile and its Gaussian curves were plotted. Scale bar, 0.5 mm.


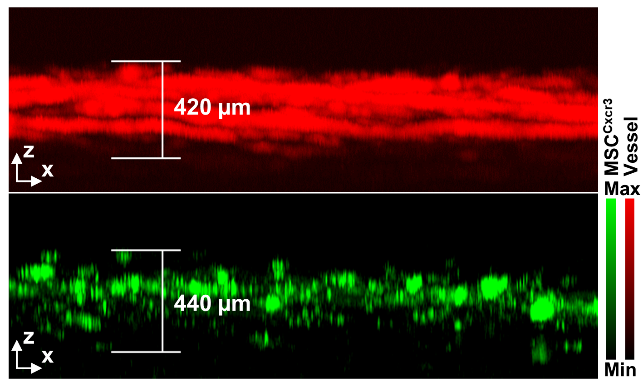


**Fig. S6** Xz projected PA image of inflamed ear (2.00 × 0.75 mm, x × z) in relation to labeled MSC^Cxcr3^ (2#) in Fig. S4-5.


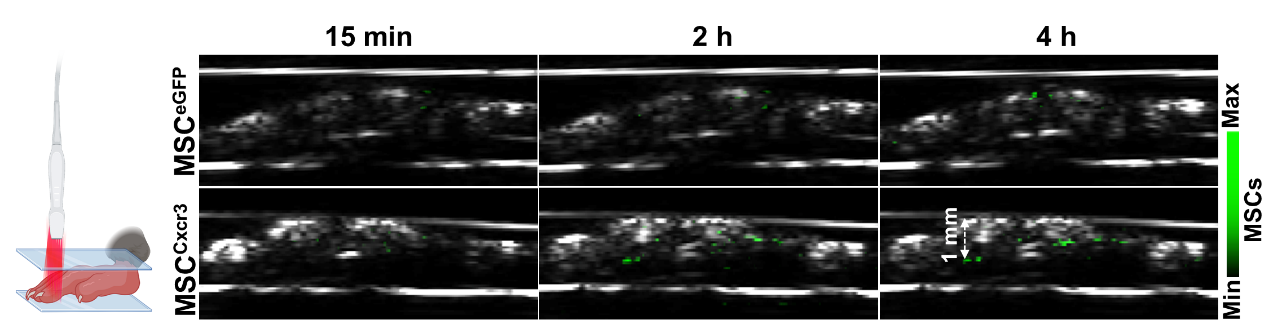


**Fig. S7** Representative PA/US overlaid images showing a more significant accumulation of TAT-CPNPs labeled MSC^Cxcr3^ than MSC^eGFP^ in the hindlimbs of rheumatoid arthritis mice model over time. A schematic illustration of PA/US dual-modal imaging was shown on the left. Images were captured 15 min, 2 h and 4 h after receiving TAT-CPNPs labeled MSC^eGFP^ or MSC^Cxcr3^ injection.
